# Perivascular macrophages in the central nervous system: insights into their roles in health and disease

**DOI:** 10.1038/s41419-025-07592-2

**Published:** 2025-04-28

**Authors:** Xiaoni Zhan, Shuying Wang, Nicholas Bèchet, Gunnar Gouras, Gehua Wen

**Affiliations:** 1 School of Forensic Medicine, China Medical University, Shenyang, Liaoning Province China; 2 Neural Plasticity and Repair Unit, Department of Experimental Medical Science, Lund University, Lund, Sweden; 3 Experimental Dementia Research Unit, Department of Experimental Medical Science, Lund University, Lund, Sweden; 4 Department of Anesthesiology, The First Hospital of China Medical University, Shenyang, Liaoning Province China; 5 Wallenberg Center for Molecular Medicine, Lund University, Lund, Sweden; 6 Department of Clinical Science, Lund University, Lund, Sweden; 7 Lund Stem Cell Center, Lund University, Lund, Sweden

**Keywords:** Cellular neuroscience, Molecular neuroscience

## Abstract

Perivascular macrophages (PVMs) are a specialized subset of macrophages situated near blood vessels in the brain. Their strategic positioning around these vessels enables them to perform key functions in immune surveillance and response to inflammation and injury. These cells are crucial for modulating the immune response within the brain, contributing to normal central nervous system (CNS) processes. In pathological conditions, the role of PVMs becomes more complex. Depending on the specific disease or injury, they may contribute to inflammation, blood-brain barrier (BBB) dysfunction, and the clearance of abnormal materials. PVMs are implicated in degenerative diseases, cerebrovascular impairment, and microhemorrhages associated with amyloid-β immunotherapy. Despite their important roles in the CNS, research on PVMs remains limited, and the mechanisms underlying their involvement in both physiological and pathological processes within the brain are not yet fully elucidated. Therefore, this review will focus on the current advancements in PVM research, including their origin, classification, roles in neuroinflammation and neuroprotection, and their potential roles as therapeutic targets for neurodegenerative diseases.

## FACTS

- PVMs are a subtype of border-associated macrophage (BAM) and serve as crucial immune cells in the brain. They can be identified based on their perivascular localization and the expression of specific markers.
- Under physiological conditions, PVMs are responsible for immune surveillance and maintaining BBB integrity, helping to sustain CNS homeostasis; in pathological states, they play complex roles in inflammation, blood-brain barrier dysfunction, and clearance of abnormal materials.
- PVMs are implicated in diseases such as Alzheimer’s Disease (AD), Parkinson’s disease (PD), stroke, and hypertension, potentially making them a notable point for therapeutic research in these conditions.

## OPEN QUESTIONS

- What do we know about PVMs?
- How do PVMs interact with other cells in the brain?
- What functions do PVMs serve under physiological conditions?
- What are the specific mechanisms of PVMs in different diseases?

## Introduction

**Fig. 1:**
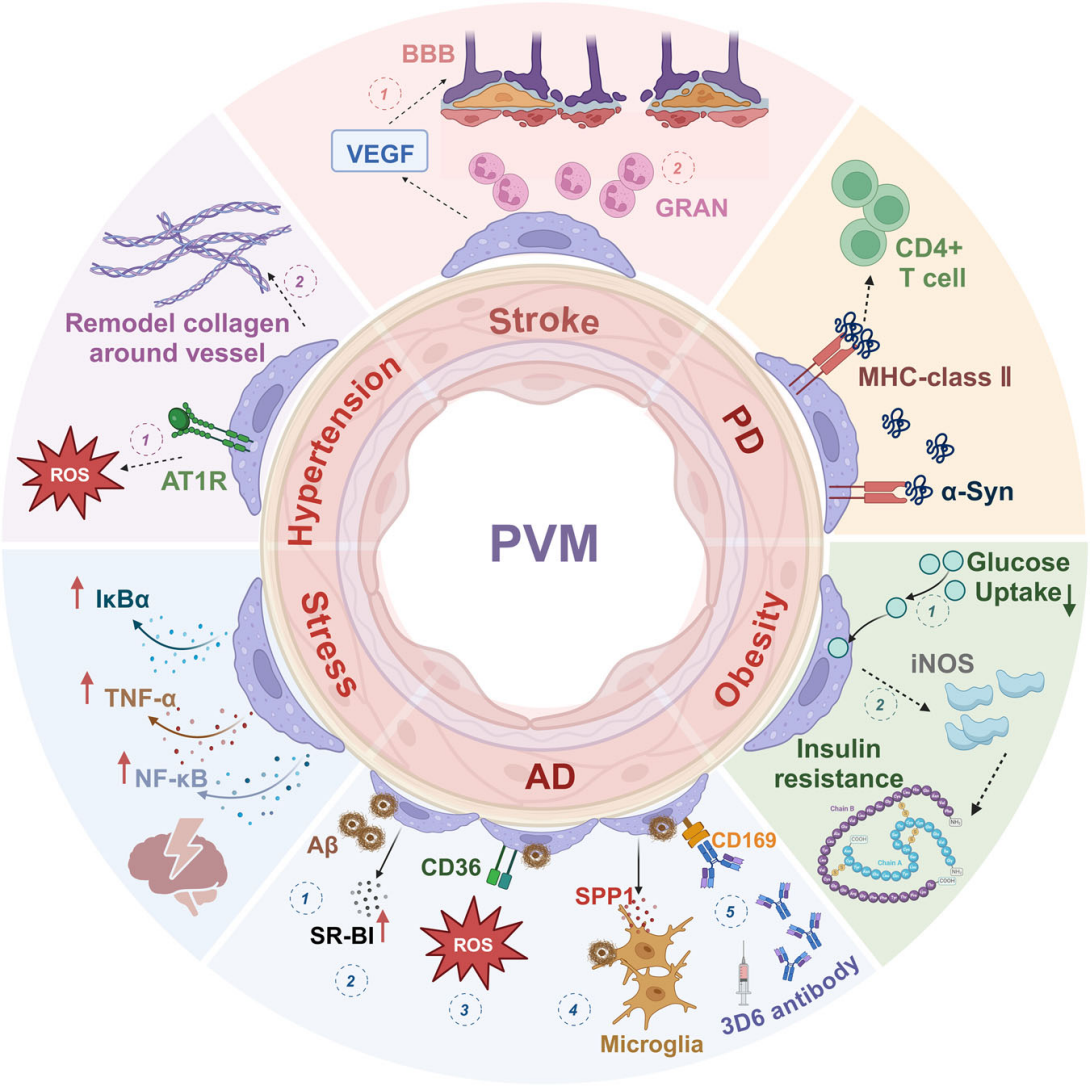
PVMs in the development and progression of various diseases. **Stroke**: 1. PVMs enhance local vascular permeability by increasing the production of vascular endothelial growth factor (VEGF) in the ischemic region. 2.PVMs, along with meningeal macrophages (MM), recruit granulocytes (GRAN) to the leptomeningeal and perivascular spaces in response to brain ischemia and inflammation. **PD**: Major Histocompatibility Complex Class II (MHC-II) expressed in BAMs, including PVMs, serve as antigen-presenting cell (APC) in the inflammation induced by α-synuclein, enhancing the recruitment and infiltration of immune cells. **Obesity**: 1. The response of PVMs to a High-Fat Diet (HFD) leads to decreased glucose uptake and compromises the integrity of the BBB. 2. inducible nitric oxide synthase (iNOS) released by PVMs leads to insulin resistance and enhances the inflammatory response. **AD**:1. PVMs help reduce Cerebral Amyloid Angiopathy (CAA) in AD by clearing amyloid-beta (Aβ) deposits around blood vessels. 2. PVMs-derived Scavenger Receptor Class B Type I (SR-BI) helps to clear Aβ. 3. CD36 in PVMs accelerates neurovascular dysfunction by increasing Reactive Oxygen Species (ROS) production. 4. Secreted Phosphoprotein 1 (SPP1) secreted by PVMs regulates the ability of microglia to phagocytose Aβ. 5. In Aβ immunotherapy, the 3D6-Aβ antibody causes blood-brain barrier disruption and microhemorrhage by activating the CD169 receptor on PVMs. **Stress**: PVMs enhance stress-induced neuroinflammation by regulating pro-inflammatory cytokine signaling, particularly through the Tumor Necrosis Factor-alpha (TNF-α), Nuclear Factor Kappa B (NFκB), and Inhibitor of Nuclear Factor Kappa B Alpha (IκBα) pathway. **Hypertension**: 1. The activation of Angiotensin II Type 1 Receptor (AT1R) on PVMs leads to the production of ROS, resulting in vascular dysfunction. 2.PVMs are involved in cerebrovascular remodeling by facilitating the production of type I collagen around cerebral arterioles. Image generated with Biorender.

Although the brain was once thought to be an immune-privileged site, a growing body of research suggests that immune cells play a crucial role in maintaining brain homeostasis and are increasingly linked to the development of brain disorders [1, 2]. Numerous studies have focused on microglia and astrocytes, demonstrating their significant influence on brain development, function, and their involvement in the pathophysiology of neurodegenerative diseases [3–6]. Moreover, certain myeloid populations have been demonstrated to play a fundamental role in maintaining brain homeostasis and disease development [7]. Non-parenchymal BAMs, such as PVMs, are a type of innate immune cell in the brain [8]. These cells are involved in brain development, the maintenance of homeostasis, and neurodegenerative diseases, such as AD [9], and PD [10, 11]. These macrophages are formed during development from embryonic precursors originating in the yolk sac and are thought to be not renewed by blood monocytes in adulthood [12]. PVMs are situated in the perivascular spaces, where they play vital roles at the brain-blood interface while remaining in direct contact with cerebrospinal fluid (CSF) [13]. Under physiological conditions, PVMs contribute to the maintenance of brain homeostasis, including facilitating BBB integrity and performing immune functions such as phagocytosis and antigen presentation [1]. Moreover, PVMs play a crucial role in various diseases. Previous studies showed that PVMs are involved in the pathology of AD [14], PD [15], stress [16], obesity [17], stroke [18], and hypertension [19] (Fig. 1). Understanding the roles of PVMs in maintaining brain homeostasis and influencing disease progression can offer valuable insights for developing future treatment strategies for the diseases. In this review, we aim to elucidate the physiological function of PVMs, as well as the alterations they undergo in various diseases. This establishes a foundation for future research endeavors focused on PVMs.

## Introduction of PVMs

### Origin and maintenance of PVMs

**Fig. 2:**
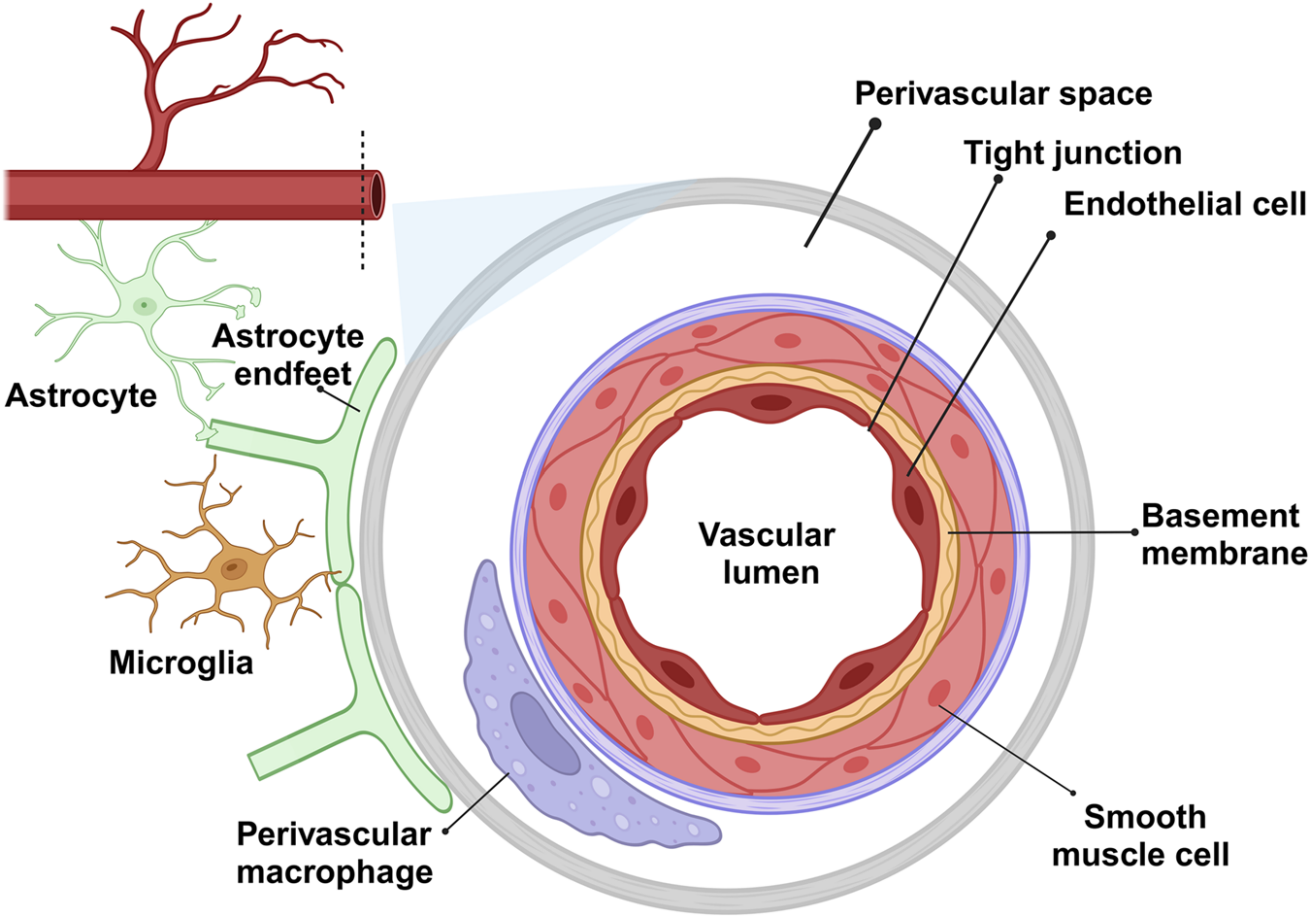
PVMs are located within the perivascular spaces, also known as VRS, which are fluid-filled compartments surrounding blood vessels in the brain. These spaces are found between the blood vessel wall (specifically the endothelium and smooth muscle layers) and the surrounding glial cells. PVMs reside close to the vasculature, interacting with the endothelial cells and the BBB, playing a key role in immune surveillance, waste clearance, and inflammatory responses in the CNS. Their strategic location allows them to regulate the movement of molecules between the blood and the brain. Image generated with Biorender.

PVMs are found most abundantly in association with pial and parenchymal vessels larger than 20 um in diameter [20]. PVMs are a distinct subset of BAMs. This category also includes meningeal macrophages (MMs) and choroid plexus macrophages (CPMs) [21, 22]. BAMs derive from prenatal progenitors located in the extra-embryonic yolk sac blood island known as erythro-myeloid progenitors (EMPs) [23]. These EMPs give rise to immature A1 macrophage progenitors, which subsequently differentiate into A2 pre-macrophage progenitors. The emergence of the initial BAM population around the developing brain coincides with the establishment of the meninges at embryonic day 9.5 (E9.5) during embryogenesis [24, 25]. During the initial stages of postnatal development, MMs undergo a continuous infiltration into the perivascular space, expanding through local proliferation [26]. The transformation of MMs into PVMs within the brain requires the assistance of specific cells, such as vascular smooth muscle cells (VSMCs). Following the formation of the Virchow–Robin spaces (VRS), the recruitment of PVMs, originating from perinatal leptomeningeal macrophages, into these spaces is dependent on the presence of vascular VSMCs [12]. VSMCs have been identified as essential for the proper distribution of PVMs during development [27, 28]. This is particularly evident in Notch3-deficient brains, where impaired and reduced arterial VSMCs correlate with a decreased population of PVMs [27]. BAMs are established prenatally and persist throughout life with limited self-renewal, except for dura mater and choroid plexus stromal macrophages, which are continually replenished by bone marrow-derived cells under homeostatic conditions [29]. Under physiological conditions, PVMs show high stability and are not thought to be replaced by mononuclear cells from the peripheral circulation [30]. A notable characteristic of PVMs is their selective distribution around arteries and arterioles in both mice and humans, while there is only a minor population of PVMs colonizing veins and venules [31]. The unique location of PVMs determines their function. PVMs in the VRS between vessel walls and parenchyma bridge peripheral signals to the CNS, regulating immune surveillance and fluid balance, which is crucial for CNS communication with the periphery [32] (Fig. 2).

### Marker proteins of PVMs

Molecularly, PVMs express specific markers including CD163, CD206, and lymphatic vessel endothelial hyaluronan receptor 1 (Lyve-1) [13]. PVMs were first characterized in 1988 by Hickey and Kimura, who termed them “perivascular microglial cells” [33]. These cells, identified by their cell surface glycoprotein ED2 expression, display an elongated morphology and are close to cerebral blood vessels. Subsequently, it was determined that ED2+ perivascular cells were distinct from microglia, as microglia lacked ED2 positivity [34]. Purification and sequencing revealed that the ED2 antigen is CD163, a membrane glycoprotein belonging to the scavenger receptor cysteine-rich (SRCR) superfamily group B [35]. CD163 is crucial in recognizing and endocytosing hemoglobin/haptoglobin complexes and actively participating in antigen presentation processes [36, 37]. CD163 is expressed in PVMs and circulating monocytes but not microglia [38]. CD206 is another widely accepted marker for PVMs and is more specific to PVMs compared to CD163 [39]. The expression of CD206 is evident in PVMs, MMs, and CPMs while absent in microglia [24]. The distinctive distribution of CD206 highlights its utility as a discerning marker, enabling the differentiation between BAM populations and microglia or infiltrating monocytes in the CNS.

Lyve-1 is prominently observed in lymphatic endothelial cells (LEC) and specific macrophages, with no expression detected on microglia [1]. In peripheral tissues, macrophage populations associated with blood vessels are identified as Lyve1^high^, while those associated with nerve bundles are characterized as Lyve1^low^ [40]. PVMs are divided into two major subtypes by their expression of Lyve-1 or MHC II, and these indicate that the two subtypes have different functions [41]. Drieu et al. identified Lyve-1^+^ PVMs located alongside α smooth muscle actin (αSMA^+^) arteries/arterioles and MHC II^+^ (antigen-presenting cells) PVMs positioned along αSMA^−^ veins/venules, highlighting the discrete functions of PVMs within the perivascular compartments of arteries and veins. They found that Lyve-1^-^ MHC II^+^ PVMs upregulated pathways that are involved in the immune response to viruses, cytokine production, cell-cell adhesion, and antigen presentation [42]. However, Lyve-1^+^ MHC II^low/neg^ PVMs were highly phagocytic and endocytic cells, expressing high levels of scavenger receptors such as CD163, Mannose Receptor C-Type 1 (Mrc1), and Macrophage Scavenger Receptor 1(Msr1). These Lyve-1^+^ PVMs exhibit phagocytic activity and are involved in hypertension and stroke [43]. Marie et al. discovered that Lyve-1^+^ PVMs display distinct patterns and proportions of association with blood vessels, influenced by both the diameter of the vessels and whether they are arterial or venous [43]. There are 3 types of Lyve-1^+^ PVMs coverage patterns: I-linear, II-intermediate, and III-circumferential. The pattern I, known as the “linear” pattern, describes a singular chain of Lyve-1 positive PVMs encircling the vessel. Pattern I was predominantly present in proximity to smaller caliber vessels, generally within the diameter range of 10–25 mm. Pattern II, referred to as the “intermediate” pattern, involves multiple Lyve-1 positive PVMs around the cross-section of vessels, and in pattern II vessels, PVMs exhibit an irregular cellular structure. Pattern II was observed in vessels ranging from 15 to 50 mm in diameter, while pattern III was linked to larger vessels ranging from 30 mm up to over 100 mm in diameter. Pattern III, labeled as the “circumferential” pattern, exhibits a greater density of coverage and an irregular cellular structure. In mice, pattern III is more abundant than pattern II and I in veins and arteries on the 11^th^ and 20^th^ days after birth [43]. Lyve-1^+^ PVMs’ dynamic distribution may relate to vessel development and aging, suggesting a role in neurodegenerative diseases, making them potential therapeutic targets for age-related conditions. Recently, single-cell sequencing has significantly improved the precision of isolating PVMs through flow cytometry [24]. By targeting cells marked by CD11b^+^CD45^high^CD206^+^, researchers achieve a highly selective and accurate isolation process. This advanced approach enhances the identification and purity of PVMs, enabling more detailed and reliable studies of their functions and roles in various physiological and pathological contexts. There are also other markers for PVMs. PVMs exhibit elevated expression of CD45, F4/80, and C-X3-C Motif Chemokine Receptor 1 (CX3CR1), while lacking the microglial-specific marker purinergic receptor Purinergic Receptor P2Y12 (P2RY12).

In conclusion, a reliable approach for identifying PVMs might involve the combined use of multiple PVM markers, alongside their specific anatomical localization and phagocytic properties. As research advances and our understanding of PVMs improves, the markers used to identify these cells are likely to change. Given that PVMs remain relatively understudied, current markers may not fully reflect their distinct features or functions. As we gain more insights into their biology, new, more specific markers will likely emerge, enabling clearer identification of PVMs and distinguishing them from other macrophage types. Here, we have summarized current data on the characteristics of BAMs and microglia (Table 1).

**Table 1:** Characteristics of brain-resident microglia and BAMs.

Cell type	Origin	Location	Turnover	Cell marker	Reference
Microglia	Yolk sac	Brain parenchyma	Self-renewal	TMEM119, P2RY12, IBA1, CD11b, MHCII, CX3CR1	[28, 109–114]
Perivascular macrophages	Yolk sac Fetal liver	Perivascular Space	Self-renewal	CD206, CD163, CD45, MHCII, LYVE1,CD38, CD169, CD36	[19, 24, 45, 114–116]
Meningeal macrophages	Yolk sac Fetal liver	Subarachnoid space and pia mater	Self-renewal	CD206, CD163, CD45, MHCII, LYVE1, CD38, CD36, CD169	[24, 29, 45, 112, 117, 118]
Choroid plexus macrophages	Yolk sac Fetal liver Bone marrow	Choroid plexus	Self-renewal; blood monocyte	CD206, CD163, CD45,MHCII, LYVE1,CD38, CD36, CD169	[24, 45, 117, 119–123]

## Interaction with other cell types

### microglia

Microglia are located within the brain parenchyma and have a cell body size of approximately 7–10 μm [21]. They function as the brain’s resident immune cells, playing a crucial role in brain development and participating in processes such as synaptic pruning, synapse maturation, and angiogenesis [44]. Microglia arise from primitive macrophages that migrate to the developing brain at embryonic day (E) 9.5, where they differentiate and contribute to neural development and homeostasis [45]. At present, the interaction between PVMs and microglia under physiological conditions remains poorly understood. However, under AD pathological conditions, SPP1 secreted by PVMs is involved in regulating the phagocytic activity of microglia within the brain parenchyma. In the brain, SPP1 expression is tightly regulated in an age-dependent and cell type-specific manner, where it participates in immune regulation and inflammatory processes by interacting with immune cells to modulate their activity and function [46]. In a recent study, a specific activation pattern of SPP1 in the perivascular space of the hippocampus in the AD mouse model was observed [47]. Microglia in the hippocampus of APP^*NL-F*^ mice exhibit significantly increased synaptic material uptake, around seven times higher than wild-type mice, with hippocampal SPP1 levels elevated threefold and primarily concentrated in PVMs [47]. Subsequent cell-sorting experiments utilizing fluorescent SPP1-expressing mice confirmed the presence of the protein in PVMs, with minimal detection in perivascular fibroblasts and none in microglia. In wild-type mice, injection of synthetic oligomeric Aβ into the ventricles led to a threefold increase in hippocampal SPP1 protein levels within 18 hours, accompanied by a fivefold increase in synaptic uptake by microglia, whereas microglia in SPP1 knockout mice showed no increase in synaptic engulfment following oligomeric Aβ injection [47]. In summary, in the AD mouse model, SPP1 secreted by PVMs regulates microglial phagocytosis of Aβ.

### VSMCs

VSMCs make up the center layer of arteries, the tunica media [48]. They are responsible for numerous physical homeostatic functions in arteries and arterioles while also contributing to the production and remodeling of the Extracellular Matrix (ECM) [49]. PVMs are characterized by their specific localization within the VRS, situated between two basal laminas: one originating from endothelial cells or the basement membranes of VSMCs and the other from the astrocytic endfeet [24, 50]. Given that PVMs are mainly situated within the VRS of arteries and arterioles containing VSMCs, there may be interactions between these two cell types. Subsequent research found that arterial VSMCs play an important role in influencing the appropriate distribution of PVMs. The function of Notch3 in VSMCs is essential for the formation of PVMs [12]. The arteries and arterioles in Notch3^−/−^ brains exhibited a significant reduction in VSMCs [51]. Importantly, in Notch3^−/−^ mice, the number of PVMs was markedly decreased compared to Notch3^+/−^ littermate controls [12]. However, mature macrophages can also reciprocally regulate VSMCs. Coculture models of macrophages and VSMCs in vitro contribute to understanding the interactions. Morisaki et al. [52]documented that macrophages enhance VSMCs proliferation by releasing platelet-derived growth factor (PDGF) in the coculture system. Another study found that interleukin 6 (IL-6) and TNF-α derived from macrophages boost the production of MMP-1 (Matrix metalloproteinase-1) by VSMCs [53].

### Circulating monocytes

The maintenance of MMs and PVMs appears not to be reliant on circulating monocytes [24]. In contrast, CPMs demonstrate relatively short-lived compared to other CNS macrophages and undergo some replenishment from circulating monocytes [24]. Under physiological conditions, PVMs exhibit slow self-renewal, and with aging, their numbers along the vasculature remain constant; monocytes in peripheral blood rarely infiltrate the brain parenchyma [41]. However, under conditions of disease or injury, circulating monocytes can differentiate into PVMs. In the spared nerve injury (SNI) mouse model, SNI leads to alterations in chemokines that attract monocytes. C-X-C Motif Chemokine Ligand 12 (CXCL12) plays a crucial role in converting monocytes into PVMs in the brain during neuropathic pain [54]. In AD model mice, circulating monocytes can infiltrate the perivascular space of the brain or surround amyloid plaques, mediating inflammatory responses [55]. Circulating monocytes specifically migrate to amyloid plaques and acquire microglial markers, indicating that newly derived macrophages are recruited to enhance microglial phagocytosis of amyloid [55]. Circulating monocytes are important in clearing plaques within the brain parenchyma, thereby contributing to the maintenance of neural health. Similarly, PVMs actively participate in the removal of perivascular Aβ deposits [56]. This clearance process helps to reduce the accumulation of Aβ around cerebral blood vessels, thereby alleviating CAA and potentially mitigating its associated pathological effects.

### Pericyte

Pericytes are perivascular cells located within the capillary wall, sharing a basement membrane with endothelial cells [57]. Pericytes serve as key regulators of neurovascular function, supporting BBB formation and maintenance, and maintaining vascular stability and structural integrity of the CNS [58]. Pericytes and PVMs are strategically positioned within the neurovascular unit (NVU) and perivascular spaces, contributing to brain endothelial cell activation and neuroinflammation [59]. Their location within the NVU facilitates close interactions between these cells and the surrounding perivascular spaces. During disease states, pericytes have been shown to migrate to the site of injury [60]. Pericytes produce a range of adhesion molecules and chemokines/cytokines that support the recruitment and migration of monocytes, T cells, eosinophils, and neutrophils [61, 62]. In a mouse model of experimental allergic encephalomyelitis (EAE), chondroitin sulfate proteoglycans (CSPGs), a family of ECM proteins, were highly enriched in inflamed perivascular cuffs [63]. These CSPGs secreted proinflammatory cytokines that activated pericytes, which subsequently facilitated the infiltration of monocyte-derived macrophages into the brain’s perivascular spaces [63, 64]. This implies that pericytes might play a role in the turnover of PVMs, though this has yet to be verified. During disease states, pericytes have been shown to migrate to the site of injury [60]. Deepak [65] discovered that in brain sections of Multiple Sclerosis (MS) patients, Platelet-Derived Growth Factor Receptor Beta (PDGFRβ^+^) cells migrated into the perivascular space. These pericytes migrate into the perivascular space rather than the brain parenchyma. We speculate that they may interact with PVMs in the perivascular space, but no studies have confirmed this yet.

## Physiological functions of PVMs

### Regulation of glymphatic drainage

The glymphatic system is a brain-wide fluid clearance pathway that facilitates the exchange of CSF and interstitial fluid (ISF). It is essential in clearing metabolic waste, including Aβ, from the CNS [66]. Past studies have indicated that the polarization and expression of aquaporin-4 (AQP4) by astrocytes are crucial for maintaining the circulatory function of the glymphatic system. The AQP4 situated on astrocytic end-feet facilitates the convective bulk movement of CSF within the interstitial spaces bordering the periarterial and perivenous pathways; abnormal polarization of AQP4 under aging and various pathological conditions disrupts the normal circulatory function of the glymphatic system [67, 68]. However, recent work showed that PVMs are pivotal for the glymphatic pathway. A recent study found that PVMs regulated CSF flow by engaging in ECM remodeling, which subsequently affects arterial pulsations [41]. CSF flow exhibits a pulsatile pattern, primarily driven by the cardiac cycle. The fact that the arterial wall moves at the same speed as the CSF suggests that arterial wall motion is the main driving force behind CSF flow [69]. When using clodronate liposomes to deplete parenchyma border macrophages, including PVMs, with no impact on the population and morphology of microglial cells and polarization of AQP4, a notable reduction of the glymphatic influx was observed by measuring the coverage of fluorescent ovalbumin (OVA) injected into the cisterna magna of mice [41]. Furthermore, the expression of ECM-associated genes (Lgals3, Col1a2, Tgfb2, Lum, Col11a1, Col8a2) was upregulated in mice depleted of PVMs. PVMs are expected to control the degradation of the ECM through the secretion of matrix metalloproteinases (MMPs), including MMP2 and MMP9, which break down the ECM produced by fibroblasts and VSMCs [41, 70]. Thus, PVMs regulate the size of the perivascular space through MMPs without affecting cerebral blood flow, thereby modulating the function of the glymphatic circulation system. Similarly, PVMs may be involved in age-related impairments of glymphatic circulation; CSF flow was found to be compromised in older mice [71]. Comparing PVMs in young (3 months old) and older (24 months old) mice revealed no significant difference in the overall number of CD206^+^ cells, however, older mice exhibited a notable reduction in Lyve1^+^ PVMs and an increase in MHCII^+^ PVMs [41]. Lyve1^+^ PVMs play a crucial role in regulating arterial motion and ECM remodeling along both large vessels and capillaries. When PVMs are dysfunctional, it leads to impaired arterial motion, ECM accumulation, and disrupted CSF flow.

### Immune surveillance

#### Antigen presentation

PVMs are thought to mediate T-cell entry into the parenchyma during neuroinflammation by MHC expression. After phagocytosis, macrophages present peptide fragments on MHC molecules, activating T-cells and subsequently triggering an inflammatory response with the release of cytokines and chemokines [72]. The MHC class I receptor on PVMs is crucial for promoting the infiltration of CD8 T cells into the brain during Theiler’s murine encephalomyelitis virus (TMEV) infection [73]. PVMs expressing MHC class II have been identified as capable of presenting antigens to lymphocytes in an experimental model of allergic encephalomyelitis [33]. Schonhoff et al. [10] discovered that BAMs, including PVMs, are crucial in facilitating neuroinflammation related to α-synuclein. Their distinctive function as APCs is essential for initiating a CD4 T cell response. Deletion of MHC II from BAM resulted in a reduction in neuroinflammatory markers, microglial activation, and CD4^+^ T cell infiltration, leading to neuroprotection against dopaminergic cell loss in the substantia nigra pars compacta (SNpc).

#### Phagocytosis

**Fig. 3:**
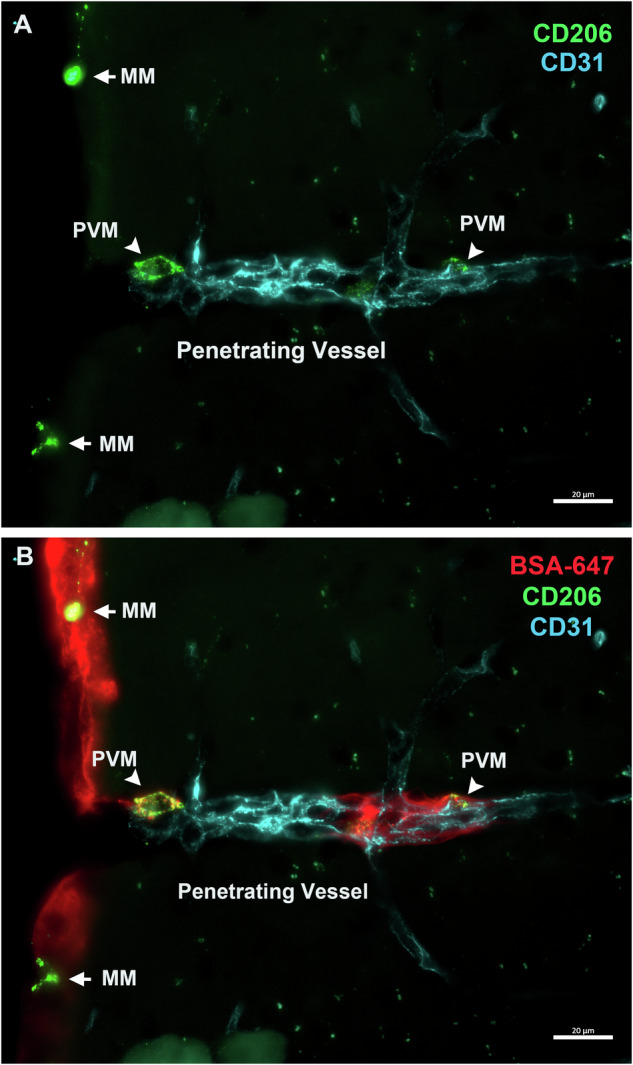
Cisterna magna injection of BSA-647 of 5-month-old C57BL/6 mouse. This is a penetrating vessel in the cortex. Here, we showed that PVMs and MMs can phagocytose BSA-647. **A** Staining with the endothelial cell marker CD31 (blue) and PVM marker CD206 (green). **B** Staining with the endothelial cell marker CD31 (blue) and PVM marker for CD206 (green), BSA-647 (red). Scale Bars = 20 μm.

PVMs engage in clearing apoptotic cell debris and removing waste materials from the ECM [59]. Their phagocytic activity contributes to the maintenance of vascular system homeostasis. The more evident manifestation of the phagocytic function of PVMs is their absorption of substances injected into parenchyma or CSF circulation. PVMs can be labeled and distinguished through cisterna magna [41, 74], intra-striatal administration [74], or intracerebroventricular (i.c.v.) injection [19] of tracers like fluorescent-labeled dextran or Albumin from Bovine Serum (BSA)-647. BSA-647 is phagocytosed by PVMs, resulting in a granular labeling pattern indicative of intracellular storage within phagosomes. Kaur et al. [74] investigated the function of PVMs in the perivascular spaces of arterioles and venules during glymphatic influx and efflux. Their results indicated that PVMs filter CSF components, participating in glymphatic clearance along arteries and veins. FITC-dextran tracer accumulation in PVMs occurred in the pial-glial periarterial spaces after intra-striatal and intra-cisternal administration. The findings of this study collectively underscore the critical role of PVMs in facilitating interstitial waste clearance during glymphatic CSF influx along arterial pathways and efflux along venous routes. Herein, we show cisterna magna injection of BSA-647 in wild-type mice to detect the phagocytic function of PVMs. We can see the PVM (green) located in the penetrating vessel (blue) and MMs (green) phagocytosing BSA-647 (red) (Fig. 3). To better study the function of PVMs, they are typically ablated in specific disease models. Liposome-encapsulated clodronate (liposome-clod), utilizing the phagocytic function of PVMs, has become a promising way to study the functionality of PVMs. Liposome-clod is injected via intracerebroventricular [32, 75] or cisterna magna [41] to deplete PVMs and MMs. Once phagocytosed by macrophages, liposome-clod functions as a cytotoxic ATP analog, disrupting mitochondrial oxygen consumption and inducing apoptosis and subsequent cell death [19, 76]. A single injection of liposome-clod, around 7 days post-administration, leads to the depletion of a significant portion of PVMs and MMs [77]. Importantly, this process has no impact on microglia and astrocytes, with a gradual recovery of these macrophages starting around the 14th day post-liposome-clod injection [75]. In various research studies, scientists have selectively ablated PVMs across different disease models to investigate and better understand their specific functions within the pathological processes. The outcomes and detailed observations from these experiments are summarized in Table 2. Despite current technological limitations, PVM ablation can be a valuable research approach, but it has many limitations. The direct pharmacological ablation of PVMs is too aggressive and does not rule out potential effects on other brain cells. With advancements in technology, better experimental strategies may emerge in the future to address this issue.

**Table 2:** PVMs in disease models.

Model	PVMs manipulation	Effect	Reference
AD	Clodronate, 5xFAD mice	Promote Aβ clearance	[41]
CAA	Clodronate, TgCRND8 mice	Promote Aβ42 clearance peptides in the vasculature	[56]
	Bone marrow chimeras, Tg2576	ROS production	[100]
	PDAPP, hTau APP KI mice	Enhance vascular permeability, plasma protein extravasation, monocyte infiltration, and microhemorrage	[9]
PD	B6.129P2(Cg)-Cx3cr1tm1Litt/J, B6.129P2(C)-Cx3cr1tm2.1(cre/ERT2)Jung/J and C57BL/6-Tmem119em1(cre/ERT2)Gfng/J mice	CD4^+^ T recruitment and antigen presentation	[10]
HFD	LysM-cre mice, LysMGFP and LysMtdT mice	Increase inducible nitric oxide synthase (iNOS) and VEGF, damage BBB integrity	[86]
Stroke	Clodronate, Sprague-Dawley rats	Increase vascular permeability, recruit granulocytes to the perivascular spaces	[18]
Hypertension	bone marrow chimeras B6.129P2-Agtr1a–/– and B6.129S-Cybbtm1Din/J mice were transplanted in C57BL/6 mice	Reactive Oxygen Species (ROS) producer	[19]
Acute restraint stress	Clodronate,Wistar Hannover rats	TNFα producer	[16]
Systemic inflammation	Clodronate, Sprague-Dawley rat	Mediate LPS-induced HPA activation	[32]

### Granulocyte chemotaxis

PVMs are essential for immunological surveillance and scavenging activities [10]. Previous studies suggested that ischemic conditions in the brain lead to the extravasation of neutrophils from leptomeningeal vessels and the accumulation of neutrophils in the perivascular spaces within the affected brain region [78, 79]. In a rat model of ischemic stroke, PVMs play a role in granulocyte recruitment. Jordi’s study showed that following brain ischemia, PVMs attract granulocytes to the perivascular spaces, resulting in neutrophil accumulation in the affected brain regions [18]. In a rat model of bacterial meningitis, PVMs release chemokines to promote leukocyte migration into the CSF, reducing the bacterial load in the CSF [80].

### Maintenance of the BBB

The BBB’s integrity is crucial for maintaining brain homeostasis. It serves as a key anatomical barrier, regulating the entry of essential components into the brain parenchyma while preventing the infiltration of pathogens and harmful blood-derived substances [81]. The integrity and function of the BBB depend on the tight junctions between brain endothelial cells, the PVMs that surround endothelial cells in the perivascular space, as well as the astrocytic endfeet enclosing the capillaries and the pericytes embedded in the capillary basement membrane [17]. Under physiological conditions, PVMs play a critical role in maintaining BBB integrity. The area postrema, a specialized brainstem region lacking tight junctions, exhibits inherently high BBB permeability. In this region, PVMs have been observed to capture serum proteins ranging from 10 to 70 kDa from the bloodstream [1, 82]. Another study found that PVMs directly limit the permeability of BBB. He et al. [83] observed that the depletion of PVMs led to an unexpected increase in the hyperpermeability of the endothelium. This hyperpermeability effect could be rescued when PVMs were reconstituted. Under pathological conditions, Machteld found that during bacterial meningitis, PVMs promote leukocyte infiltration by regulating BBB permeability [80]. This may be due to the activation of PVMs in the early stages of meningitis, leading to the production of mediators (cytokines) that induce leukocyte migration [80]. Through in vivo two-photon microscopy and electron microscopy in an angiotensin-II-induced hypertensive mouse model, Monica et al. [84] identified that arterioles and venules larger than 10 μm are most vulnerable to increase BBB permeability. The depletion of PVMs effectively reduces this increased BBB permeability, specifically in arterioles. Based on the above studies, we can infer that PVMs may have a bidirectional regulatory role in BBB permeability under both physiological and pathological conditions.

### Regulation of nutrient uptake

PVMs may influence brain metabolism by modulating cellular nutrient uptake. A single-cell atlas of mouse brain macrophages revealed the increased expression of genes related to lipid metabolism and the storage of cholesterol in PVMs [29]. Jais et al. [85] documented a transient upregulation of vascular endothelial growth factor (VEGF) expression by PVMs in the hypothalamus of mice subjected to HFD. Meanwhile, in HFD-fed conditions, PVMs exhibit the presence of intracellular lipid droplets, thereby mitigating the risk of excessive lipid accumulation in the extracellular space of the hypothalamus [86]. In the HFD model, PVMs affect both lipid metabolism and glucose uptake. This model shows a reduction in glucose transporter 1 (GLUT1) expression in vascular endothelial cells, resulting in decreased glucose entry into the CNS [85]. In response to this reduction, PVMs release VEGF, which stimulates an increase in GLUT1 expression in endothelial cells, thereby restoring glucose uptake [85].

## PVMs and diseases

### Activation of the hypothalamic-pituitary-adrenal (HPA) axis and systemic response to stress

PVMs have been implicated in triggering the activation of the HPA axis. The activation of the HPA axis and induction of fever are associated with systemic inflammation, processes that necessitate the presence of prostaglandin E2 (PGE2) [87]. Systemic administration of interleukin-1β (IL-1β) or lipopolysaccharide (LPS) leads to an elevation in the expression of cyclooxygenase-2 (COX-2) and microsomal prostaglandin E2 synthase (mPGES), which is one of the key enzymes involved in the synthesis of PGE2 [88]. In IL1β-triggered systemic inflammation of wild-type rats, depleting PVMs suppressed the elevation of endothelial COX-2 and PGE2, as well as the activation of the HPA axis. Conversely, in LPS-induced inflammation, the depletion of PVMs intensified this reaction [32]. Hence, PVMs may exert dual roles in modulating the activation of the HPA axis during systemic inflammation, depending on the nature and complexity of the inflammatory trigger.

PVMs are also modulators of stress-induced neuroinflammation and oxidative stress [16]. After i.c.v. administration of liposome-clod to rats for PVM depletion, the rats were then placed in a transparent plastic tube for 6 hours to restrict their movement. In this acute stress model, the authors underscore the intricate involvement of PVMs in pro-inflammatory cytokine signaling. Notably, The depletion of PVMs is associated with concurrent changes in several signaling pathways and cellular responses: (1) a reduction in pro-inflammatory cytokine signaling, specifically decreasing TNF-α levels; (2) downregulation of the JAK/STAT pathway, accompanied by an increase in IL-6 receptor expression under stress conditions; (3) alterations in TLR4 signaling, affecting nearly all elements in the pathway studied, with a notable reduction in the acute stress-induced expression of MyD88; (4) decreased expression of NFκB, IκBα [16]. Taken together, PVMs may exert a promotive role in the inflammatory response elicited by acute stress.

### PVMs and cerebrovascular diseases

#### Hypertension

Hypertension is a major contributing factor to both stroke and dementia in the elderly, primarily due to its disruption of blood flow to the brain [89]. Strategically positioned around brain vessels, PVMs are important in perivascular drainage and cerebrovascular flexibility and function as scavengers and surveillance cells under physiological state [72]. Previous research has shown that circulating macrophage infiltration into vascular walls contributes to the development of hypertension by promoting vascular inflammation and endothelial dysfunction [90, 91]. Recent work found that PVMs are also significantly involved in the development of hypertension; PVMs are robust producers of ROS in hypertension [19]. During hypertension onset, vascular angiotensin-II can breach the BBB, activating AT1R on PVMs, which in turn produce significant levels of ROS, leading to vascular dysfunction [19]. PVMs also exert a substantial influence on changes in BBB permeability during the development of hypertension. In a hypertension model of transgenic BPH/2 J mice, BBB permeability was compromised by 8 weeks of age, preceding cognitive impairment at 12 weeks. Notably, Depleting PVMs at this stage restored BBB integrity and ameliorated cognitive deficits [84].

PVMs might also contribute to the advancement of hypertensive conditions. PVMs are also involved in the cerebrovascular remodeling process associated with hypertension. This remodeling involves generating type I collagen around cerebral arterioles [92]. In the hypertension model of SHRSP/Izm rats, PVMs surrounding cerebral small vessels express Col1a1 mRNA, thereby facilitating the production of type I collagen [92]. This process leads to collagen deposition around the cerebral small vessels and contributes to atherosclerosis during the pathological process of hypertension. However, the detrimental effects of PVMs in the progression of hypertension still require further research.

#### Stroke

Stroke remains the third-leading cause of death and disability combined in the world in 2022 [93]. Ischemic stroke, which accounts for approximately 62.4% of all stroke cases, is the most common type of stroke [72]. In an adult male Sprague-Dawley rat model of stroke by intraluminal occlusion of the right middle cerebral artery (MCAo) followed by reperfusion, Pedragosa and colleagues [18] provided evidence for a role of PVMs and MMs increasing vascular permeability, facilitating granulocyte recruitment, and contributing to neurological dysfunction in the acute phase of ischemic stroke. PVMs and MMs recruit granulocytes to the leptomeningeal and perivascular spaces in reaction to brain ischemia and increased inflammation. Acute production of VEGF is associated with increases in vascular permeability [94]. PVMs and MMs increase vascular permeability by increasing VEGF production in the ischemic region of stroke. The depletion of PVMs and MMs in rats led to a decline in the expression of Vegfa mRNA and its secreted protein isoform, VEGF164 [18]. Therefore, PVMs may promote the increased local vascular permeability associated with ischemic stroke, thus contributing to its pathological progression.

### Neurodegenerative disease

#### AD

AD is the most common neurodegenerative condition globally and modern therapeutic strategies have been notably shaped by the following key neuropathological features: deposition of Aβ in extracellular spaces, development of intraneuronal neurofibrillary tangles (NFTs), and neuroinflammation [95]. The involvement of PVMs in Aβ plaque clearance and disease progression has gained widespread attention in recent years due to their phagocytic function. In the 5xFAD mouse model, intracerebroventricular injection of liposome-clod depleted PVMs led to increased Aβ plaque deposition, particularly in the cerebral cortex and amygdala [41]. This suggests that although PVMs are located around blood vessels, they may contribute to the clearance of Aβ within the brain parenchyma. Similarly, in another AD model of APP/PS1 mouse, there is a significant discrepancy in the density of Lyve-1 positive PVMs between brain regions with plaque count and concentration [43]. Regions rich in plaques are associated with reduced Lyve-1^+^ PVM densities, whereas areas with relatively higher Lyve-1 levels exhibit fewer plaques. The mechanism is still unknown, but it is reasonable to consider that Lyve-1^+^ PVMs contribute to plaque clearance and affect plaque abundance. Another study found that PVMs are crucial in removing waste products including Aβ from the brain tissue through scavenger receptors. SR-BI mediates the response of PVMs and regulates Aβ-related pathology and CAA in the AD mouse model [96]. SR-BI serves as a high-density lipoprotein receptor, controlling the removal of cholesterol from peripheral tissues to the liver [97]. In the AD brain, SR-BI has been detected in astrocytes and VSMCs, and it has been reported to facilitate the adhesion of microglia to fibrillar Aβ. Kalliopi et al. [98] found that SR-BI also exists in PVMs. PVMs-derived SR-BI are seen as important in Aβ clearance [98]. PVMs aggregate near regions of Aβ deposition and increase their expression of SR-BI. Crossing an AD mouse model with a transgenic mouse line deficient in SR-BI led to elevated Aβ deposition in the brain, indicating that SR-BI expression by PVMs facilitates their Aβ clearing capacity. Reduction in SR-BI impairs the response of PVMs to Aβ and increases the expression of phagocytic receptors on PVMs, such as CD206 and CD163 [8, 98]. This increase suggests a compensatory response to the loss of SR-BI. Up to 90% of AD patients are associated with the presence of CAA, which involves the accumulation of Aβ in the blood vessels of the cortex and leptomeninges [99]. Another study focused on the role of PVMs in removing CAA. In the TgCRND8 mouse model of AD (overexpression of human KM670/671NL Swedish and V717F Indiana mutant APP), the depletion of PVMs exacerbated CAA severity, resulting in a significant increase in thioflavin S-positive cortical blood vessels and selective accumulation of Aβ42 peptides in the vasculature [56].

In summary, the role of PVMs in AD appears to primarily involve promoting the clearance of Aβ. However, in addition to this protective function of removing Aβ, PVMs can also accelerate the neurovascular dysfunction observed in regions where Aβ deposits occur. Deletion of CD36 from PVMs improved neurovascular function, reduced ROS production, and decreased Aβ_40_ in blood vessels [100]. This led to a significant reduction in arteriolar smooth muscle cell damage and CAA without affecting parenchymal amyloid plaques. Targeting CD36 on PVMs could potentially preserve the beneficial effects on Aβ clearance while mitigating the deleterious effects of ROS production in vascular structure and function. These findings reveal a detrimental role of PVMs in cerebrovascular Aβ accumulation mediated by CD36. PVMs exhibit a dual role in AD, exerting both protective and detrimental effects. From one perspective, they aid in clearing Aβ aggregates, potentially slowing disease progression. Conversely, they can intensify neuroinflammation, possibly accelerating disease advancement.

#### PD

The characteristic neuropathological features of PD include the degeneration of dopaminergic neurons within the SNpc and the development of intraneuronal protein aggregates of insoluble α-synuclein [101]. Past researches have primarily focused on the role of microglia and astrocytes in clearing these synaptic/nuclear proteins within the brain [102, 103]. A recent study found that BAMs, including PVMs, play a crucial role in coordinating neuroinflammatory and neurodegenerative reactions in the pathology of PD. BAMs are pivotal in triggering α-synuclein-induced neuroinflammatory responses by recruiting peripheral immune cells and restimulating antigens [10]. BAMs are important in facilitating the restimulation of CD4^+^ T cells essential for α-synuclein-mediated local cytokine production and entry into the brain parenchyma. Notably, BAMs exhibited high expression of genes associated with T cell recruitment, such as Ccl5 and Ccl10, antigen processing, and presentation like H2-Aa, Cd74, and Cd274. This suggests that antigen presentation, specifically by BAM, is essential for the initiation of the inflammatory response and subsequent neurodegeneration induced by α-syn. Furthermore, selective depletion of BAMs resulted in a reduction in neuroinflammation, including microglial activation and the infiltration of peripheral immune cells, emphasizing the crucial role of BAMs in α-synuclein-mediated neuroinflammation. Given their unique location, ability to initiate lymphocyte chemotaxis, and involvement in the remodeling of the parenchymal extracellular matrix, BAMs could potentially provide a new direction for PD therapy in the future.

### Aβ immunotherapy

Aβ immunotherapy is a novel treatment approved for AD, preclinical experiments showed that antibodies can diminish Aβ accumulation in the brain and reverse memory impairments [104–107]. PVMs have been shown to help in the clearance of vascular Aβ in mouse models of AD [56, 98, 100]. Importantly, Aβ immunotherapy in patients supports the involvement of PVMs in Aβ phagocytosis and clearance within cerebral blood vessels, highlighting their role in the response to Aβ immunotherapy [108]. In contrast, the damaging effects of Aβ immunotherapy on blood vessels are also associated with PVMs. The 3D6-Aβ antibody induces the formation of immune complexes at vascular amyloid deposits, activating CD169^+^ PVMs and leading to increased plasma protein leakage and a higher incidence of microhemorrhages in PDAPP mice [9]. CAA deposits recruit and activate CD169^+^ PVMs, which in turn enhance vascular permeability, plasma protein extravasation, and monocyte infiltration, thereby increasing the risk of microhemorrhages [9]. Furthermore, these findings suggest that the activation of PVMs is linked to the amplification of the local inflammatory environment and disrupted remodeling of the ECM surrounding vascular Aβ deposits. It is possible that regulating the CD169 receptor on PVMs could be utilized to prevent or reduce microhemorrhages and BBB damage caused by Aβ immunotherapy and applied to clinical Aβ immunotherapy.

### Metabolic syndrome associated with obesity

PVMs in the hypothalamus upregulate the expression of iNOS and VEGF after prolonged consumption of HFD [17]. This PVM’s response to HFD decreases glucose uptake and compromises the integrity of the BBB, making it more permeable. As a result, this increased permeability allows for a greater influx of lipids and inflammatory cells into the hypothalamic tissue. This influx can lead to inflammation and other metabolic disturbances, further exacerbating the negative effects on brain function and overall metabolic health. Lee et al. [86] showed that inhibiting hypothalamic iNOS, which is primarily released by PVMs, led to a reduction in inflammatory markers in HFD-fed mice. Furthermore, this inhibition improved glucose intolerance and reduced systemic insulin resistance in obese mice. PVMs exhibit intracellular lipid droplets when exposed to HFD conditions due to their phagocytic activity [86]. This helps to limit the accumulation of lipids in the hypothalamic extracellular space, which would otherwise be harmful to neurons and other cells. Although these initial findings are promising, there remains a shortage of studies investigating the mechanisms through which PVMs contribute to obesity-related hypothalamic dysfunction. Further research is needed to confirm the role of PVMs in this pathological process.

## Conclusion and prospects

In conclusion, PVMs are essential for maintaining homeostasis within the CNS and responding to a range of pathological conditions. Their strategic location surrounding blood vessels allows them to fulfill critical roles, including immune surveillance, preservation of the BBB, and enhancement of neuroprotection. While current studies have revealed the significance of PVMs in neuroinflammation and neurodegenerative diseases, there are still important gaps in our comprehension of their precise mechanisms of action and how they interact with other immune cells in the brain. Future research should concentrate on the interactions between PVMs and other cells in the CNS, such as neurons, microglia, and astrocytes. Gaining a deeper understanding of these interactions will provide insight into how PVMs influence CNS immune responses and the function of the NVU, particularly in pathological conditions. Further research is required to clarify the role of PVMs in neurodegenerative diseases. A deeper investigation into their contributions to disease pathogenesis, including their involvement in neuroinflammation, Aβ clearance, and blood-brain barrier maintenance, may provide insights into novel therapeutic strategies. Exploring these research areas will deepen our understanding of the diverse roles of PVMs in CNS physiology and pathology, potentially leading to new therapeutic approaches for treating CNS disorders.
